# Anisotropic optical response of optically opaque elastomers with conductive fillers as revealed by terahertz polarization spectroscopy

**DOI:** 10.1038/srep39079

**Published:** 2016-12-23

**Authors:** Makoto Okano, Shinichi Watanabe

**Affiliations:** 1 Department of Physics, Faculty of Science and Technology, Keio University, 3-14-1 Hiyoshi, Kohoku-ku, Yokohama, Kanagawa 223-8522, Japan

## Abstract

Elastomers are one of the most important materials in modern society because of the inherent viscoelastic properties due to their cross-linked polymer chains. Their vibration-absorbing and adhesive properties are especially useful and thus utilized in various applications, for example, tires in automobiles and bicycles, seismic dampers in buildings, and seals in a space shuttle. Thus, the nondestructive inspection of their internal states such as the internal deformation is essential in safety. Generally, industrial elastomers include various kinds of additives, such as carbon blacks for reinforcing them. The additives make most of them opaque in a wide spectral range from visible to mid-infrared, resulting in that the nondestructive inspection of the internal deformation is quite difficult. Here, we demonstrate transmission terahertz polarization spectroscopy as a powerful technique for investigating the internal optical anisotropy in optically opaque elastomers with conductive additives, which are transparent only in the terahertz frequency region. The internal deformation can be probed through the polarization changes inside the material due to the anisotropic dielectric response of the conductive additives. Our study about the polarization-dependent terahertz response of elastomers with conductive additives provides novel knowledge for *in situ*, nondestructive evaluation of their internal deformation.

Because of their various advantages such as light weight, mass producibility, and low cost, polymeric materials – including plastics, elastomers, and organic polymers – are of great importance^1^,^2^,^3^,^4^. Recently, numerous practical applications have been suggested and extensively investigated, and the improvement of polymeric materials in terms of durability has enabled metallic components to be replaced with polymeric components^1^. In particular, polymeric composite materials have attracted attention because of their additional functionality – such as conductivity, durability, and so on – that results from including certain additives^1^,^2^,^3^. In fact, polymeric composites consisting of elastomers and various additives represent one of the most widely used classes of polymeric materials in modern society^3^. To date, carbon materials have been widely used as a reinforcing filler in elastomers. (For instance, the tire is a popular elastomer that is reinforced by carbon fillers.) Given their crucial role in modern industry, it is essential to evaluate the degradation and internal states of polymeric composite materials in a safe manner.

In order to measure and evaluate polymer-based materials, various methods – such as X-ray diffraction (XRD), optical measurements, and nuclear magnetic resonance spectroscopy – have been developed and conducted^5^,^6^,^7^. Although XRD has been commonly used for evaluating molecular orientation, this approach is usually insensitive to the internal states of thick materials and the orientation of the amorphous states^5^. On the other hand, there are several advantages to using optical techniques in measuring polymeric materials, which enable us to achieve *in situ* nondestructive and noncontact inspection^8^,^9^,^10^. However, since most of the practical polymer-based materials are opaque for visible light, one cannot assess the internal information of polymer-based materials by means of optical techniques with visible light.

Terahertz time-domain spectroscopy (THz-TDS) is a powerful tool for characterizing the internal optical responses of optically opaque polymer-based materials^11^,^12^,^13^,^14^,^15^,^16^,^17^,^18^,^19^,^20^,^21^,^22^,^23^,^24^,^25^,^26^. Because the photon energy of terahertz waves is relatively small in comparison to that of visible light, they possess high transmittance, which enables us to access the internal states of opaque polymeric materials. Indeed, the strong influence of carbon-based additives on the optical properties of polymeric composite materials within the terahertz frequency region has been reported using terahertz spectroscopy^13^,^15^,^16^,^17^,^18^,^19^,^20^,^23^. Moreover, very recently, polarization-sensitive (PS) measurements using terahertz waves have been drastically improved by the efforts of many researchers^27^,^28^,^29^,^30^,^31^,^32^,^33^,^34^. In particular, polarization spectroscopy for polymer materials has been utilized for evaluating the molecular orientation of polymer materials, which is strongly related to their mechanical, optical, and electrical properties^6^,^7^,^8^,^9^,^10^,^35^,^36^,^37^. A better understanding of the internal optical responses involved in the molecular orientation of opaque polymer-based materials using PS THz-TDS techniques would provide detailed insights into the nature of the materials in question, especially in terms of intermolecular interactions and the effects of additives.

In this study, we report on the large optical anisotropy of loaded and unloaded fluoroelastomer (FKM) with carbon black (CB) fillers as revealed by PS THz-TDS with a transmission configuration; we also discuss the physical origin of the optical anisotropy. The frequency dependence of the complex refractive index indicates that the anisotropic orientation of CB aggregates is the origin of the anisotropic optical responses, including birefringence. We measure the external stress dependence of birefringent properties in the FKM samples with different angles between the slow optic axis and the stretching direction. As the external stress increases, the FKM samples oriented at an angle of 90° with respect to the stretched direction demonstrate the following characteristic behaviors: the disappearance of birefringence and the rotation of the angle of the slow optic axis to the stretching direction. The rotation of the slow optic axis is well reproduced by Monte Carlo (MC) simulations, which are based on the dominant impact of the orientational distribution of the CB aggregates on the birefringence. To further investigate the anisotropic optical properties, the dielectric functions of the FKM samples parallel and perpendicular to the slow optic axis are evaluated and fitted by the Maxwell Garnett (MG) theory with the anisotropic dielectric functions of the CB fillers. The well-reproduced fitting supports the claim that the anisotropic shape and the anisotropic dielectric responses of the CB aggregates in the terahertz frequency region are the origin of the optical anisotropy of the FKM samples.

## Results and Discussion

### Characterization of mechanical properties

**Figure 1:**
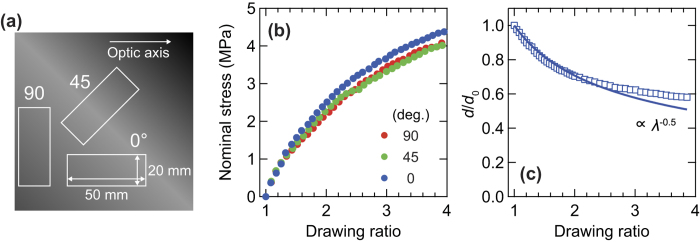
Sample orientation and mechanical properties of the FKM samples. (**a**) Orientation of the rectangular specimens cut from the FKM sheet. (**b**) Nominal stress-strain curves for variously oriented FKM samples. (**c**) Thickness ratio of loaded to unloaded 0°-oriented FKM samples for various drawing ratios. Solid curve represents the fitted curve.

The FKM samples with different orientation angles used for the characterization of their mechanical properties were cut from the FKM sheet as shown in Fig. 1a (see Methods for details). Figure 1b,c show the nominal stress and thickness ratio, *d*/*d*_0_, as a function of the drawing ratio (DR), *L*/*L*_0_, where *d* and *L* are the sample thickness and length, respectively, at different external stresses applied by the translation stages, and *d*_0_ and *L*_0_ are those values under unloaded conditions. As the DR increases, the external stress becomes larger and then tends to be saturated. These behaviors are typical in elastomers, and are well-described by the Mooney-Rivlin model^38^,^39^. No significant difference in the stress-strain curves among three samples is observed, indicating that the direction of the slow optic axis has a weak impact on the mechanical properties of FKM samples. The thickness ratio of the 0°-oriented FKM samples shows a gradual nonlinear decrease as the DR increases, as shown in Fig. 1c. The dependence is almost proportional to the inverse square root of the DR below DR = 3, meaning that the Poisson’s ratio of the FKM samples is 0.5, which is a typical value of an elastomer. On the other hand, above DR = 3, the thickness ratio does not follow the inverse square root dependence of the DR. Therefore, we focus on the optical properties below DR = 3 in order to consider the simple deformation regime with a Poisson’s ratio of 0.5.

### Transmission spectra from terahertz to visible regions

**Figure 2:**
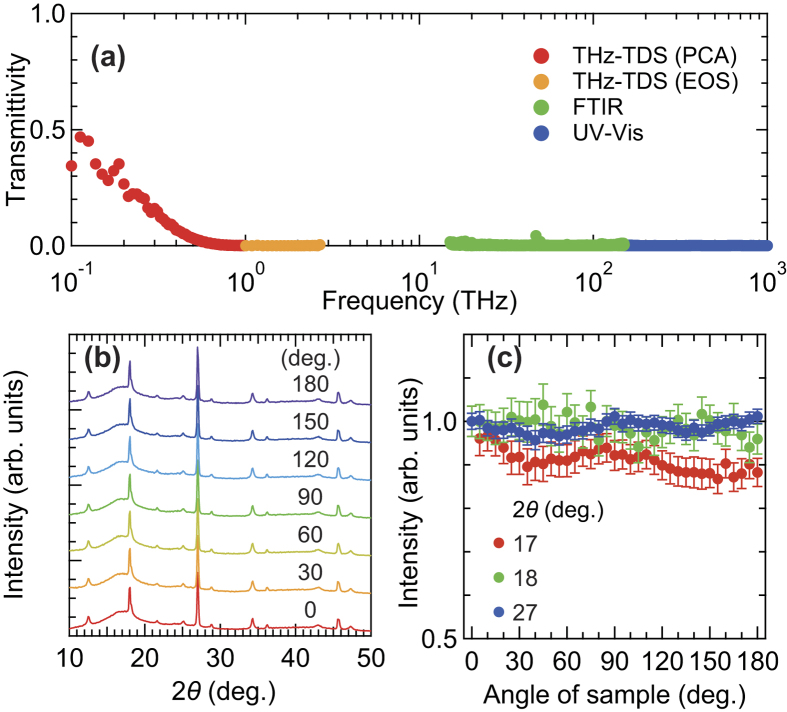
Fundamental characterization of the FKM samples. (**a**) Transmittance spectrum of FKM samples from terahertz to UV regions. (**b**) The sample-angle-dependent XRD intensities in unloaded FKM samples as a function of the incident angle of the X-ray. (**c**) Sample orientation dependence of spectrally integrated XRD intensities at 2*θ* = 17° (red), 18° (green), and 27° (blue).

Figure 2a shows the transmittance spectra in a wide spectral range from 0.1 to 1000 THz measured by the two THz-TDS systems (see Methods for details), Fourier transform infrared (FTIR) spectrometry, and spectrophotometry (UV-Vis). The absence of a transmission spectrum from 2.7 to 15 THz is due to the difficulty of terahertz generation in this region. It is clearly found that there is no transmission of FKM samples above 1 THz. The strong absorption above 1 THz can be attributed to the conductive CB fillers, as reported previously^17^. Thus, it is suggested that terahertz spectroscopy below 1 THz is useful for investigating the optical responses inside an elastomer containing CB fillers.

### Sample orientation dependence of XRD spectra

Figure 2b shows XRD signals as a function of the incident angle of the X-rays at different sample orientations ranging from 0° to 180°. When the sample orientation is 0°, the X-ray is incident normal to the slow optic axis of the FKM sample. The broad peaks around 2*θ* = 17° and 40° can be attributed to the FKM component^40^. On the other hand, many sharp peaks originate from additives or crystalline FKM components. The sample orientation dependence of the spectrally integrated intensity at various XRD peaks is plotted in Fig. 2c. These intensities are normalized at 0°. The peak at 17° due to the FKM shows small fluctuations, as do the other peaks. It can be clearly seen that the intensity of FKM signals shows no significant periodical dependence on the sample orientation.

### Birefringent properties of the FKM samples under unloaded conditions

**Figure 3:**
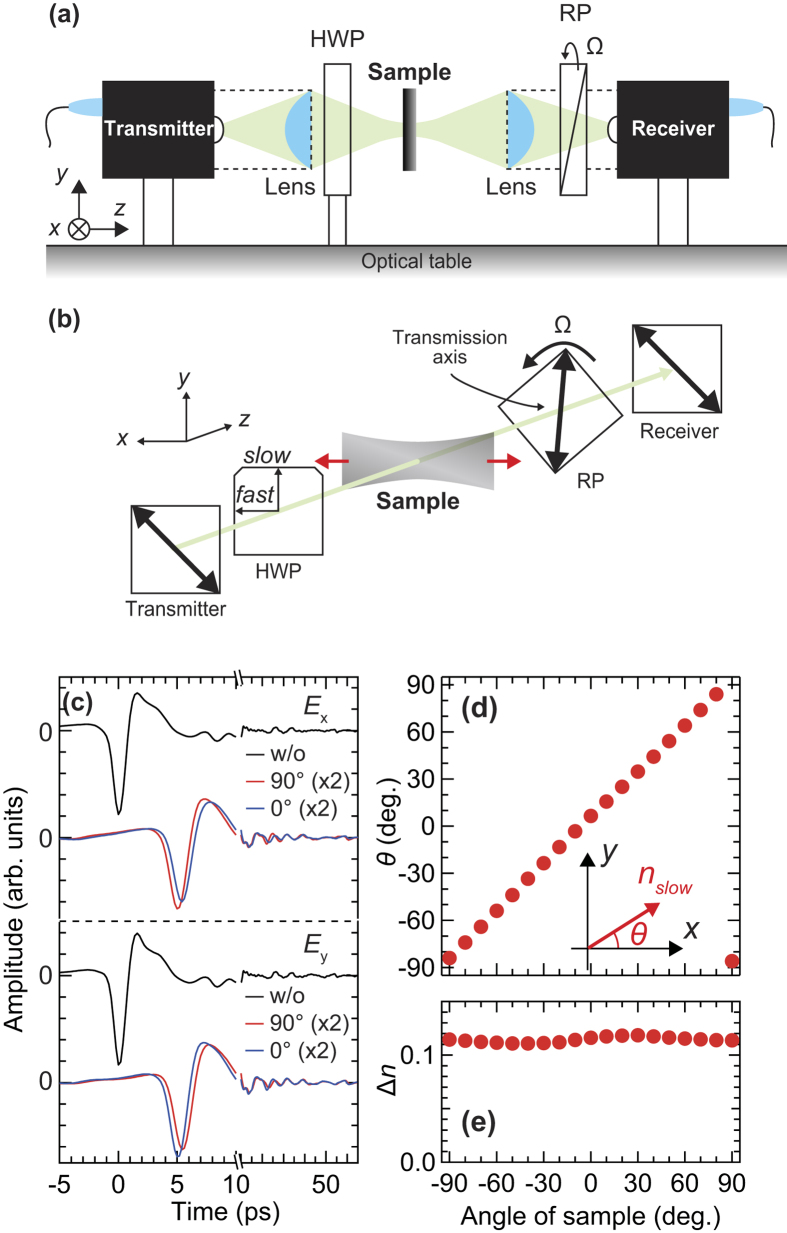
Experimental setup and results of the PS THz-TDS. (**a**) Experimental setup for PS THz-TDS with two commercial photoconductive antennas. (**b**) Schematic diagram of each optical component along with the direction of terahertz wave propagation. (**c**) Time-domain terahertz waveforms without (black curves) and with FKM samples with different sample orientations (red and blue curves). The upper and lower panels correspond to the *x* and *y*-components of the terahertz electric field. Sample orientation dependence of (**d**) the angle of the slow optic axis with respect to the *x*-axis and (**e**) the birefringence in the unloaded FKM sample. The uncertainty in the evaluation procedure is smaller than the plotted symbols.

To evaluate the birefringent properties, i.e., the angle of the slow optic axis with respect to the *x*-axis, *θ*(ω), and the degree of birefringence, Δ*n*(ω), which is derived from the phase difference of the transmitted terahertz waves between the slow and fast optic axes, Δ(ω), in unloaded FKM samples in the terahertz frequency range, we carried out the PS THz-TDS measurements. The experimental setup and configuration of the PS THz-TDS system with a rotation polarizer polarimetry are shown in Fig. 3a,b (see Methods for details). In order to measure the sample orientation dependence of the optical responses in the FKM samples, we rotated the FKM samples with a mechanically rotating mount. Based on the PS THz-TDS measurement, we found that *θ*(ω) is almost independent of frequency in the FKM samples. In this work, thus, we do not assume a frequency dependence on *θ*.

Figure 3c shows the *x*- and *y*-components of the terahertz electric field waveform without samples and with samples, where the angles between the slow optic axis of the samples and the *x*-axis are 0° (blue curves) and 90° (red curves). With the FKM sample, both components of the terahertz time-domain waveforms are shifted compared with those obtained without the samples because of the refractive index of the samples. In addition, the *x (y*) polarization component of terahertz wave with the FKM sample at an angle of 0° (90°) is slightly retarded from the component measured at an angle of 90° (0°), indicating the existence of the birefringence of the unloaded FKM samples. This indicates that the particular direction of the FKM samples always shows a large refractive index for terahertz waves with respect to its orthogonal direction.

Interestingly, we found the anisotropic optical properties of the FKM samples even under unloaded conditions. Figure 3d,e show the experimentally evaluated values of *θ* and Δ*n* averaged from 0.2 to 0.3 THz at different sample orientation angles with respect to the *x*-axis. As the sample orientation changes, *θ* shows proportional changes, whereas Δ*n* shows no significant change. This indicates the existence of an in-plane uniaxial optical anisotropy of the FKM sample. It is noteworthy that the degree of birefringence of the FKM samples (Δ*n* ≈ 0.1) within the terahertz frequency region is quite large compared to the typical value (Δ*n* ≈ 1 × 10^−3^) observed in elastomers with visible light^6^.

To shed light on the origin of the large birefringence in the FKM, we also measured other rubber materials with and without CB (see Supplementary Information for details). Experimental studies have pointed out the large influence of carbon materials on the optical properties of polymer composites within the terahertz frequency region^13^,^15^,^16^,^17^,^18^,^19^,^20^,^23^. Indeed, rubbers with CB – such as a styrene-butadiene rubber – show large birefringence (as do the FKM samples), while the birefringence of a natural rubber without CB is one order of magnitude smaller than that of the FKM samples (see Supplementary Fig. S3). Thus, we concluded that the conductive CB filler plays a dominant role in the anisotropic internal optical responses involved in the birefringence of elastomers within the terahertz frequency region.

**Figure 4:**
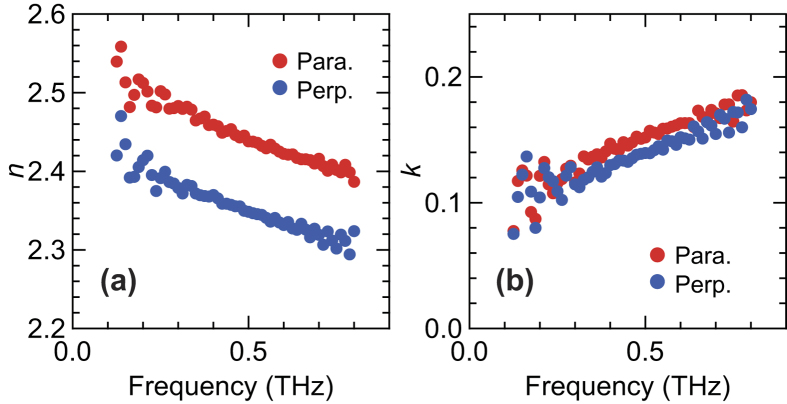
Anisotropic complex refractive index spectra of the FKM sample. (**a**) Real and (**b**) imaginary parts of complex refractive index in FKM samples. Red and blue circles correspond to the values in the parallel and perpendicular directions, respectively, with respect to the slow optic axis.

In order to further elucidate the origin of the anisotropic optical responses observed only in elastomers with CB, we evaluated the complex refractive index spectra of the unloaded FKM samples using PS THz-TDS measurements. Figure 4a,b show the real (*n*) and imaginary (*k*) parts of the complex refractive index of the FKM sample parallel and perpendicular to the slow optic axis of the sample. In Fig. 4a, both spectra monotonically decrease with increasing frequency, and the difference between the two directions is insensitive to frequency. On the other hand, the extinction coefficient *k* increases with frequency, corresponding to the monotonic increasing of the absorption coefficient. Both clearly show anisotropic properties.

The anisotropic optical responses in polymer composites with carbon nanotubes have been investigated within the terahertz frequency region^41^. According to the literature, the anisotropy is caused by the orientation of the carbon nanotubes in the host polymeric materials; more specifically, the carbon nanotubes have a high aspect ratio, which results in anisotropic optical responses. On the other hand, the shape of CB is usually considered to be spherical, which is isotropic. However, it is well known that the CB particles aggregate in the rubbers, and that the aggregates form an anisotropic shape^42^. Thus, we concluded that the orientation of anisotropic conductive CB aggregates plays a dominant role in the anisotropic optical responses of elastomers within the terahertz frequency region. This means that the collective shape of conductive particles governs their internal optical responses, rather than the shape of each particle at the terahertz frequency region. This mechanism is importantly different from conventional photoelasticity in elastomers investigated using visible light, in which the orientation of the elastomer molecule is the origin of the birefringence^6^. This difference in the origin of birefringence is responsible for the difference in the degree of birefringence, resulting in the large birefringence within the terahertz frequency range.

### External-stress-induced birefringent properties in FKM samples

As mentioned above, we found that the orientation of CB aggregates has a strong impact on the anisotropic optical properties of FKM samples. It has been demonstrated that by stretching the samples, the orientation direction of inclusion can be modulated mechanically^43^,^44^. Thus, we can obtain deep insight into the nature of the anisotropic optical properties in the FKM samples by measuring the mechanical stress dependencies of the optical responses.

**Figure 5:**
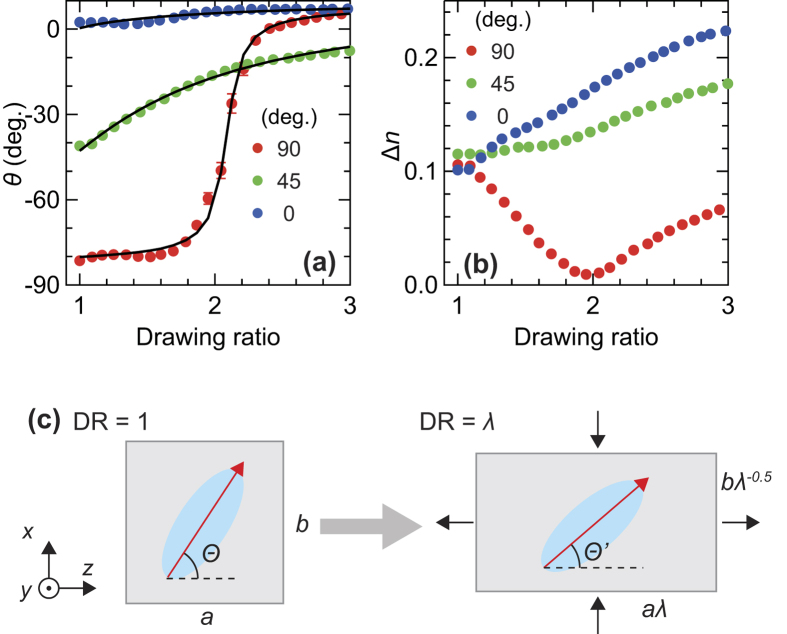
Drawing ratio dependence of the anisotropic optical properties of the FKM sample and the MC model for uniaxial deformation. (**a**) The angle of the slow optic axis with respect to the *x*-axis and (**b**) birefringence in variously-oriented FKM samples at different DRs. Solid curves in (**a**) represent the calculated results by MC simulations with *σ* = 0.21. (**c**) Schematic model of reorientation of CB aggregates by affine uniaxial deformation in FKM samples. Red arrows represent the major axis of anisotropic CB aggregates.

Figure 5a,b show *θ* and Δ*n* averaged from 0.2 to 0.3 THz versus the DR. To derive Δ*n* from the obtained Δ(ω) at each DR, we divided Δ(ω) by the thickness, which was calculated based on a Poisson’s ratio of 0.5. In these measurements, we extended the FKM samples and then fixed their length during the measurements. We always measured the center of the extended samples at different external stress conditions. In the 0°-oriented FKM sample, as the external stress increases, *θ* is almost unchanged, and Δ*n* monotonically increases. These behaviors are considered to be the result of the orientation of CB aggregates to the extended direction. By way of contrast, in the 90°-oriented FKM sample, *θ* remains around the initial angle below DR ≈ 1.6, and then gradually rotates to approximately 0° around DR = 2, which means that the slow optic axis becomes parallel to the drawing direction. Simultaneously, at DR = 2, Δ*n* of this sample becomes almost zero and changes from decreasing to increasing. The birefringence of the 45°-oriented FKM sample increases monotonically with DR, as expected intuitively by the results of the other FKM samples oriented with different angles. On the other hand, *θ* in the 45°-oriented FKM sample shows a gentle dependence on the DR compared with the 90°-oriented FKM samples. Note that *θ* of the 0°- and 90°-oriented FKM samples do not converge to 0° at DR = 3, because there is a small discrepancy between the *x*-axis and the stretched direction due to the misalignment of the polarization direction angle of the photoconductive antenna (PCA) in the receiver.

### Monte Carlo simulations for the macroscopic orientation of the CB fillers

In order to interpret the DR dependence of *θ* based on the effects of the orientational distribution of the CB aggregates, we performed MC simulations to reproduce the experimental results in the FKM samples. First, we assumed that all CB aggregates were rigid^45^,^46^ and uniform uniaxial ellipsoids for simplicity, as the uniaxial anisotropy is generally represented by the uniaxial ellipsoids. As shown in Fig. 5c, the angles between the major axis of CB aggregates, in which the refractive index is the largest, and the stretching direction (*z*-axis in Fig. 5c) at the initial and final states are defined as *Θ* and *Θ*’, respectively. Second, we assumed that the deformation of the samples occurs affinely with a Poisson’s ratio of 0.5^44^ while the length of each ellipsoid does not change (according to the first assumption that the CB aggregates are rigid^45^,^46^). Under the second assumption, the change in angle of each ellipsoid as a result of the extension can be described as follows^44^: 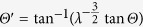, where λ is the drawing ratio. Third, the average direction of the major axis of all ellipsoids corresponds to the slow optic axis (*θ*). If one end of the major axis is at the center of the unit sphere, then the orientational distribution of the ellipsoids is determined by the distribution of the spherical angles *Θ* and *ϕ*. In this simulation, to reproduce the experimental results, we assume that the distribution of *ϕ* is uniform whereas the distribution of cos*Θ* has a normal distribution with standard deviation *σ*. Under these assumptions, we calculate the orientational distribution at various DR values and then determine the average direction of the major axis of all ellipsoids – i.e., the angle of the slow optic axis – through diagonalization of the tensor order parameter^47^. The detailed calculation procedure is described in the Supplementary Information.

The black curves in Fig. 5a represent the MC simulation results with 1,000,000 samples. Three experimental results are well reproduced by the calculation with the same initial orientational distribution of the ellipsoids (*σ* = 0.21) by only changing the initial orientation angle. The calculated results are slightly shifted in order to reproduce the experimental results, corresponding to the correction of the misalignment of the experimental setup as mentioned above. Indeed, the experimental results of the 0°-, 45°-, and 90°-oriented FKM samples are reproduced by the calculation under initial orientation angles of −9°, −52°, and −88°, respectively. The good agreement between the experimental and calculated results constitutes the solid evidence that the anisotropic optical properties of the FKM samples are governed by the orientational distribution of the anisotropic CB aggregates.

It is worth noting that the orientational distribution of the anisotropic CB aggregates is strongly related to the deformation of the host elastomer, and that its optical anisotropy due to the orientational distribution of the anisotropic CB aggregates is easily determined by the PS THz-TDS measurements. Moreover, it has been found that the orientation of anisotropic conductive additives such as single-walled carbon nanotubes also cause the optical anisotropy within the terahertz frequency region^41^. For these reasons, we believe that PS THz-TDS is a powerful tool in the *in situ* nondestructive evaluation of the internal deformation of polymeric composite materials with the anisotropic conductive additives through the anisotropic optical responses.

### Anisotropic dielectric function in loaded FKM samples

**Figure 6:**
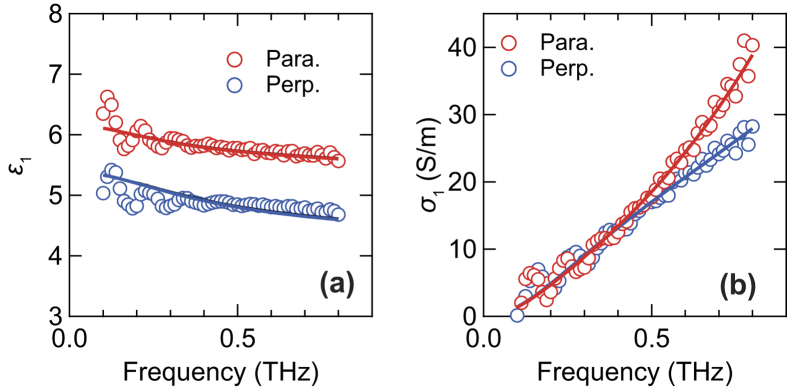
Sample-orientation-dependent dielectric function and conductivity of the FKM sample. Frequency dependence of the real part of (**a**) dielectric function and (**b**) conductivity at DR = 3, where the FKM sample was expanded along with the parallel direction of the slow optic axis. Red and blue symbols correspond to the components parallel and perpendicular to the direction of the slow optic axis, respectively. Solid curves represent the fitted curve calculated by the MG model.

In order to understand the anisotropic optical responses of the FKM samples in terms of the electric responses of the CB fillers, we evaluated the real part of the dielectric function, *ε*_1_(*ω*), and the conductivity, *σ*_1_(*ω*), in the 0°-oriented FKM samples at DR = 3, as shown in Fig. 6a,b. According to the MC simulation, most CB aggregates are oriented parallel to the stretching direction (see Supplementary Fig. S5d) at DR = 3. The values of *ε*_1_(*ω*) and *σ*_1_(*ω*) for the direction parallel to the slow optic axis (i.e., extended direction) are larger than those for the other direction, which is related to the large birefringence of the FKM samples. In fact, *σ*_1_(*ω*) shows similar behaviors as the absorption spectrum of CB^17^, resulting from the fact that the CB dominates the dielectric response within the terahertz frequency region.

To further understand the anisotropic optical properties of the FKM samples, we fit the experimental results with the theoretical model for mixed materials. Indeed, the FKM sample is considered as the mixture of CB aggregates and the host FKM matrix. Based on the experiments in other elastomers without CB, the mechanical-stress-induced anisotropy of host rubbers has less impact on the optical properties within the terahertz frequency region (see Supplementary Fig. S3). Thus, the effective dielectric responses of the FKM sample are composed of the anisotropic dielectric responses of the CB aggregates and the isotropic dielectric responses of the host rubber materials. We adopted the MG theory in order to take into account the anisotropy of the dielectric functions^48^. Because the CB density in the FKM samples is below the percolation threshold, the MG theory is applicable in considering the dielectric response of the samples.

The effective dielectric function in the MG theory, *ε*_*MG*_, can be expressed as follows^48^:


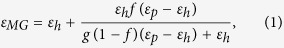


where *ε*_*h*_ and *ε*_*p*_ are the dielectric functions of the host materials and the CB aggregates, respectively, *f* is the volume fraction of the CB, and *g* is the depolarization factor. Here, we supposed that *ε*_*h*_ is real and constant, corresponding to weak absorption and independence of frequency, and that *ε*_*p*_ is described by the Drude-Smith (DS) model^49^, which is utilized for inhomogeneous media. In the DS model with a single scattering approximation, the complex dielectric function, 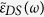, can be expressed as follows:


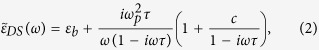


where *ε*_*b*_ is the background dielectric constant, *τ* is the scattering time, *ω*_*p*_ is the plasma frequency, and *c* is a coefficient ranging from −1 to 0, reflecting the delocalization of carriers. We assumed that the anisotropy of the dielectric function is due to the anisotropic shape of CB aggregates and the anisotropy of *ε*_*p*_(*ω*); the former was taken into account as a depolarization factor. In the case of the uniaxial ellipsoids with an aspect ratio of 1.3, which was evaluated from the SEM measurements (see Supplementary Fig. S1), the depolarization factors parallel and perpendicular to the major axis were approximately 0.27 and 0.365, respectively^48^. Moreover, in the case when all ellipsoids are fully oriented, the dielectric function of each direction is easily described by replacing *ε*_*p*_(*ω*) with *ε*_*//*_(*ω*) or *ε*_⊥_(*ω*), as previously reported^50^. In the fitting, we set *f* to 0.2 based on the SEM measurements. The fitting parameters are summarized in Table 1. By changing only *τ* (107 and 58 fs for *ε*_*//*_ and *ε*_⊥_, respectively), *ε*_1_(*ω*) and *σ*_1_(*ω*) are simultaneously well reproduced under parallel and perpendicular directions, as shown in the solid curves in Fig. 6a,b. This indicates that the anisotropic optical response of the FKM samples is due to the anisotropic shape of the CB aggregates and their anisotropic dielectric responses. Because the dielectric function of each CB particle is considered to be isotropic, the anisotropic dielectric function is mainly due to the anisotropic shape of the CB aggregates. Indeed, the difference in *τ* means that the scattering time for the direction of the major axis is larger than that for other directions, which might be related to the anisotropic shape of the CB aggregates. The anisotropic dielectric function obtained by the fitting also supports our claim that the collective shape of the CB particles influences the optical responses within the terahertz frequency region.

Finally, we briefly mention the characteristics of PS terahertz spectroscopy compared with other evaluation methods. We performed the conventional method – i.e., the stress-strain, and the wide-frequency-range transmission measurements. The comparison between terahertz spectroscopy and conventional experiments indicates that PS THz-TDS is more sensitive to the internal anisotropic properties of the elastomer with conductive fillers. Indeed, as shown in Fig. 1b, we cannot observe the anisotropy of the FKM samples in terms of the mechanical properties. Moreover, as shown in Fig. 2a, the transmission above 1 THz is very small, meaning that it is difficult to access the internal states of thick polymer composites with conductive fillers using IR and visible light. In fact, the microwave can be used to detect the anisotropic electric responses caused by the CB fillers^51^. The THz-TDS has some advantages over the microwave technique, such as higher spatial resolution and *in situ* inspection without the waveguide. Thus, since the polymer composites with conductive fillers are widely used with increasing demand, our *in situ*, nondestructive technique is of primary importance in fundamental and applied polymer science.

## Conclusions

We revealed the anisotropic optical properties of the FKM samples with CB by using PS THz-TDS measurements. The large birefringence of the FKM samples was observed at various sample orientations. By comparing against rubber materials without CB, we were able to ascribe the origin of the large birefringence to the conductive CB fillers. The external stress dependence of the birefringent properties of the FKM samples with an angle between the slow optic axis and the stretching direction of 90° showed characteristic features: namely, the degree of birefringence was diminished, and the angle of the slow optic axis rotated to the stretching direction. The dependence of the slow optic axis of variously oriented FKM samples on the DR can be explained by the MC simulation, which takes into account the orientation of the anisotropic CB aggregates. In addition, it was found that the anisotropic dielectric function of the FKM samples, which is linked to the optical anisotropy, is caused by the anisotropic shape and the anisotropic dielectric function of the CB aggregates. These results indicate that the anisotropic shape of the CB aggregates plays an important role in the anisotropic optical properties of elastomer composites. Our findings suggest that deformation in polymer composite materials can be evaluated from the degree of birefringence using nondestructive terahertz polarization measurements.

## Methods

### Sample preparation

We used a commercially available FKM sheet (V-100) purchased from Togawa Rubber with dimensions of 300 mm × 300 mm and a thickness of 1 mm as a sample. The FKM sheet includes conductive CB fillers. For the stress-strain and PS THz-TDS measurements, the FKM sheet was cut into a rectangular specimen with a short axis of about 20 mm and a long axis of about 50 mm as shown in Fig. 1a. We prepared three differently oriented samples, which were set with a 0°, 45°, and 90° angle between the long axis and the slow optic axis of the FKM samples that was determined by the PS THz-TDS spectroscopy as described below. In this paper, these samples are labeled as the 0°, 45°, and 90°-oriented FKM samples, respectively. On the other hand, the FKM samples for XRD, FTIR, and UV-Vis measurements had dimensions of about 20 mm × 20 mm.

### Transmittance measurements

FTIR was performed using an IR Affinity-1S (Shimadzu Corp.) from 15 to 150 THz, and the transmission measurements from the infrared to visible regions were carried out using UV-3600 Plus (Shimadzu Corp.) from 150 to 1000 THz. In addition, we performed two different THz-TDS measurements using a commercially available THz-TDS system based on a PCA (T-ray 5000, Advanced Photonix. Inc.) from 0.1 to 1.0 THz and a homemade electro-optic sampling (EOS) system based on a (111) ZnTe crystal for terahertz generation and a *c*-cut GaSe crystal for terahertz detection from 1.0 to 2.7 THz.

### XRD measurements

We conducted one-dimensional XRD spectroscopy using a commercially available instrument (D8 advance, Bruker Corp.). The spot size of the XRD measurements was evaluated to be approximately 1 mm × 16 mm, which was smaller than the samples.

### Stress-strain measurements

In the stress-strain measurements and the PS THz-TDS measurements under external stresses, we fixed both short axes of three variously oriented rectangular specimens on the translation stages, and then extended them along with their long axes by moving the stages. The external stress was measured using a force gauge, which was fixed on the one stage. The sample length and the sample thickness were measured at the center of the sample using a vernier caliper and a micrometer, respectively.

### Polarization-sensitive THz-TDS measurements

Figure 3a shows the experimental setup of the PS THz-TDS system, which was based on rotation polarizer polarimetry^30^,^52^. We used a commercially available high-speed THz-TDS system (T-ray 5000, Advanced Photonix. Inc.) with a measurement time of 1 ms and a frequency resolution of 12.5 GHz as a transmitter and receiver of the terahertz wave using PCAs. The terahertz pulse emitted from the transmitter was focused on the FKM samples using an aspherical Teflon lens with a working distance of 76.2 mm after passing through a half-wave plate (HWP) optimized at 0.6 THz. The terahertz pulse passing through the samples was collected by another aspherical Teflon lens with the same focal length, and was then focused on the receiver after passing through a rotating wire-grid polarizer (WGP) (EWG40-I, Origin Corporation, indicated as RP in Fig. 3a,b). The WGP was attached to a commercial hollow-shaft motor (HM2853E18HA, Technohands Co., Ltd.) with a rotating frequency of Ω/(2π) = 40 Hz. We defined the *x*-axis as the direction that is tilted −45° with respect to the polarization direction of the PCA in the receiver as shown in Fig. 3b. Namely, the *x*-axis is approximately parallel to the optical table. The positive *z*-direction is defined as the propagation direction of the terahertz wave. The spot size on the sample was measured by the knife-edge method, and was estimated to be below 3.5 mm from 0.2 to 2.0 THz.

The experimental scheme of the polarization measurement is depicted in Fig. 3b. The detected frequency-domain amplitude of the terahertz electric field, 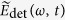, modulated by the RP with a rotating speed of Ω/(2π) can be described as follows (see Supplementary Information for details):


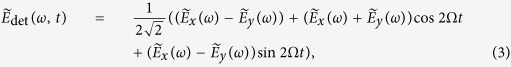


where 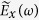 and 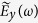 are the complex frequency domain amplitudes of the terahertz electric field for the *x*- and *y*-directions, respectively, before passing through the RP, and *t* is time. The systematic error in our experimental configuration due to the finite extinction ratios of the RP and the PCA in the receiver is considered to be negligibly small^30^. From the two orthogonal components of terahertz waves obtained by analyzing 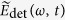, we calculate the Stokes vector **S**(ω), which represents the frequency-dependent polarization state (see Supplementary Information for details). The angle of the slow optic axis, *θ*(ω), and the phase difference of the transmitted terahertz waves between the slow and fast optic axes, Δ(ω), of the sample are determined by analyzing the change in the Stokes vector from the condition without the sample to the condition with the sample^53^,^54^,^55^. The detailed methods of determining *θ*(ω) and Δ(ω) are described in the Supplementary Information. The degree of birefringence Δ*n*(ω) of the sample is calculated from Δ(ω) by using the following relation: 
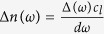
 where *c*_*l*_ is the speed of light and *d* is the thickness of the sample. All measurements as described above were carried out at room temperature.

## Additional Information

**How to cite this article**: Okano, M. and Watanabe, S. Anisotropic optical response of optically opaque elastomers with conductive fillers as revealed by terahertz polarization spectroscopy. *Sci. Rep.* **6**, 39079; doi: 10.1038/srep39079 (2016).

**Publisher's note:** Springer Nature remains neutral with regard to jurisdictional claims in published maps and institutional affiliations.

## Supplementary Material

Supplementary Information

## Figures and Tables

**Table 1:** Fitting parameters of dielectric function and conductivity with the MG theory.

	*c*	*τ* (fs)	*ε*_b_	*ε*_h_	*ω*_p_ (THz)
‖	−0.73	107	7	3.2	9.5
⊥		58			
