# Supplementary material for: Anisotropic optical response of optically opaque elastomers with conductive fillers as revealed by terahertz polarization spectroscopy
Source: Sci Rep. 2016 Dec 23;6:39079. doi: 10.1038/srep39079 (PMC5180234; doi:10.1038/srep39079)
Supplement: Supplementary Information [file srep39079-s1.pdf]

Supplementary information

Anisotropic optical response of optically opaque elastomers with conductive fillers as revealed by terahertz polarization spectroscopy

Makoto Okano* & Shinichi Watanabe

Department of Physics, Faculty of Science and Technology, Keio University, 3-14-1 Hiyoshi, Kohoku-ku, Yokohama, Kanagawa 223-8522, Japan

Characterization with scanning electron microscope (SEM): SEM was performed using an INSPECT S50 (FEI company). In order to avoid charging of the measured sample during the SEM measurements, the FKM samples were covered with a 6 nm thick osmium tetroxide coating. In addition, we carried out the SEM measurements under low vacuum (~0.5 mbar) and low electron acceleration voltage (10 kV). Figure S1a shows the SEM image of the cross section of the FKM sample. In the SEM measurements, the conductive material shows as a bright region (conductive additives). The insulating material is observed as a dark region (FKM material).

To evaluate the volume fraction of the conductive additives in the FKM samples, we binarized the image using a thresholding technique, the result of which is shown in Fig. S1b. This technique increases the contrast between the two materials in the sample so they can be more effectively quantified. In Fig. S1b, the white and black regions represent the conductive additives and the elastomer, respectively. Small white areas appear to anisotropic shapes as expected by the polarization-sensitive terahertz

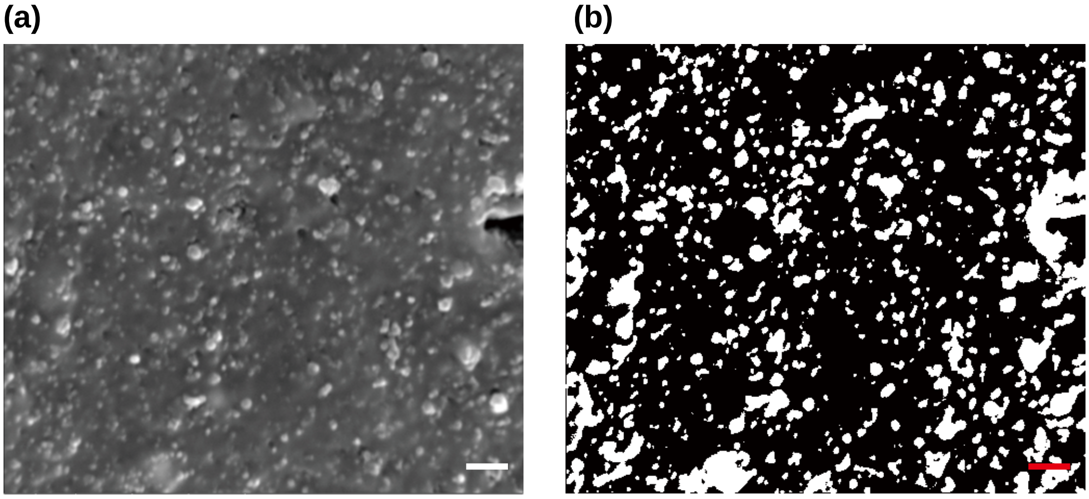

Figure S1: (a) SEM and (b) the corresponding binarized images of the cross section of the FKM sample. The slow optic axis determined by the PS THz-TDS is parallel to the vertical direction in (a) and (b). The scale bars are 1 μm.

time-domain spectroscopy (PS THz-TDS). Based on the ratio of the white to the black areas, the volume fraction of the conductive additives is estimated to be approximately 0.2. We estimate the aspect ratio of conductive additives to be ~1.3 by averaging those of the 35 ellipsoidal white spots. Note that the anisotropic orientation of the additives is difficult to evaluate from the SEM image.

Polarization sensitive (PS) THz-TDS measurements: For PS THz-TDS measurement, the two orthogonal components of the terahertz electric fileds need to be accurately evaluated. We used rotating analyzer (polarizer) polarimetry for fast and simultaneous measurement of the two components of the terahertz wave [S1]. The experimental configuration with a rotating polarizer is shown schematically in Figs. 3a,b. We explain how we obtain the x and y components of the terahertz electric field from the configuration in Fig. 3 using the Jones matrix representation.

We define the x-axis as the direction that is tilted −45° with respect to the polarization direction of the photoconductive antenna in the receiver. The terahertz wave propagates to the positive z-direction (see Fig. 3a). We set the polarization direction of the photoconductive antenna in the transmitter with an angle of 45° with respect to the x-axis, resulting in that the terahertz pulse emitted from the transmitter is linearly polarized with an angle of 45° with respect to the x-axis. The fast and slow axes of the HWP are set parallel to the x- and y-axes, respectively. The HWP is optimized for 0.6 THz. Pulses at 0.6 THz are linearly polarized at an angle of 135°. Waves that are not at 0.6 THz will be elliptically polarized by the HWP, with a frequency-dependent ellipticity.

In our PS THz-TDS system based on rotating polarizer polarimetry (see Fig. 3b), the wire-grid polarizer (WGP) is rotated with a rotation frequency of $\Omega/(2\pi)$ = 40 Hz. The detected frequency-domain amplitude spectrum of the terahertz pulse,$\tilde{E}_{det}(\omega)$ is described using the Jones matrix representation;

$$\begin{pmatrix} \tilde{E}_{det}(\omega) \\ 0 \end{pmatrix} = \mathbf{PR}(\theta_{\mathrm{det}})\mathbf{R}(-\Omega t)\mathbf{PR}(\Omega t) \begin{pmatrix} \tilde{E}_x(\omega) \\ \tilde{E}_y(\omega) \end{pmatrix},$$

where θ_{det} is the polarization angle of photoconductive antenna in the receiver with respect to the x-axis, $\tilde{E}_x(\omega)$ and $\tilde{E}_y(\omega)$ are the frequency-domain Fourier components of the terahertz wave in the x and y direction incident on the rotating polarizer, respectively, and t is time. $\mathbf{R}(\theta)$ is 2 × 2 rotation matrix and $\mathbf{P}$ is the Jones matrix of a polarizer, which are given by

$$\mathbf{R}(\theta) = \begin{pmatrix} \cos\theta & -\sin\theta \\ \sin\theta & \cos\theta \end{pmatrix}, \ \mathbf{P} = \begin{pmatrix} 1 & 0 \\ 0 & 0 \end{pmatrix}.$$

As it is well known that the photoconductive antenna has a linear polarization sensitivity [S2], the photoconductive antenna of the receiver can be described as **P** in

terms of the Jones matrix representation. In this configuration, since the effect of the finite extinction ratios of the rotating polarizer and the photoconductive antennas in the receiver on the systematic error is small [S1], we considered those as an ideal polarizer.

In this work, as we set θ_{det} to 45°, $\tilde{E}_{det}(\omega)$ is given by

$$\begin{pmatrix}\tilde{E}_{det}(\omega)\\ 0\end{pmatrix} = \frac{1}{\sqrt{2}}\begin{pmatrix}1 & 0\\ 0 & 0\end{pmatrix}\begin{pmatrix}1 & -1\\ 1 & 1\end{pmatrix}\begin{pmatrix}\cos^2\Omega t & -\cos\Omega t\sin\Omega t\\ -\cos\Omega t\sin\Omega t & \sin^2\Omega t\end{pmatrix}\begin{pmatrix}\tilde{E}_x(\omega)\\ \tilde{E}_y(\omega)\end{pmatrix}$$
$$= \frac{1}{\sqrt{2}}\begin{pmatrix}\tilde{E}_x(\omega)\cos^2\Omega t + \left(\tilde{E}_x(\omega) - \tilde{E}_y(\omega)\right)\cos\Omega t\sin\Omega t - \tilde{E}_y(\omega)\sin^2\Omega t\\ 0\end{pmatrix}.$$

Therefore, the detected amplitude of the complex Fourier components are simply described by

$$\tilde{E}_{det}(\omega) = \frac{1}{2\sqrt{2}}\left(\left(\tilde{E}_x(\omega) - \tilde{E}_y(\omega)\right) + \left(\tilde{E}_x(\omega) + \tilde{E}_y(\omega)\right)\cos 2\Omega t + \left(\tilde{E}_x(\omega) - \tilde{E}_y(\omega)\right)\sin 2\Omega t\right).$$

$\tilde{E}_x(\omega)$ and $\tilde{E}_y(\omega)$ are obtained by analyzing the amplitudes of $\cos 2\Omega t$, $A_{\cos 2\Omega t}$, and $\sin 2\Omega t$, $A_{\sin 2\Omega t}$ as represented below:

$$\begin{cases}\tilde{E}_x(\omega) = \frac{A_{\cos 2\Omega t} + A_{\sin 2\Omega t}}{2}\\ \tilde{E}_y(\omega) = \frac{A_{\cos 2\Omega t} - A_{\sin 2\Omega t}}{2}\end{cases}.$$

A control experiment was performed with a WGP at an angle of 45° relative to the *x*-axis at the sample position for adjustment of the phase of motor. The angle of the WGP is identical to the polarization angle of the photoconductive antenna in the receiver.

From the *x*- and *y*-components of the terahertz electric field, we can evaluate the three normalized Stokes parameters, S_1, S_2, and S_3, that represent the polarization state in the following equations [S3].

$$S_1 = \frac{\tilde{E}_x(\omega)\tilde{E}_x^*(\omega) - \tilde{E}_y(\omega)\tilde{E}_y^*(\omega)}{\tilde{E}_x(\omega)\tilde{E}_x^*(\omega) + \tilde{E}_y(\omega)\tilde{E}_y^*(\omega)},$$
$$S_2 = \frac{\tilde{E}_x(\omega)\tilde{E}_y^*(\omega) - \tilde{E}_y(\omega)\tilde{E}_x^*(\omega)}{\tilde{E}_x(\omega)\tilde{E}_x^*(\omega) + \tilde{E}_y(\omega)\tilde{E}_y^*(\omega)},$$

$$S_3 = \frac{i\left(\tilde{E}_y(\omega)\tilde{E}_x^*(\omega) - \tilde{E}_x(\omega)\tilde{E}_y(\omega)\right)}{\tilde{E}_x(\omega)\tilde{E}_x^*(\omega) + \tilde{E}_y(\omega)\tilde{E}_y(\omega)}.$$

In the next section, we describe the method by which the birefringent properties can be evaluated using a change in the Stokes parameter based on the Poincaré sphere representation.

Evaluation of the birefringent properties using the Poincaré sphere representation:

We explain a procedure for analyzing the angle of the slow optic axis (θ) and the phase difference of the transmitted terahertz waves (Δ) between the slow and fast optic axes from changes in the polarization state using the Poincaré sphere representation. The Poincaré sphere is a powerful tool to visualize change in polarization states of light passing through optical elements and experimental samples [S4]. In the Poincaré sphere representation, each polarization state of light corresponds to a different coordinate on the sphere. For instance, linearly polarized light has coordinates across the equator that vary as a function of orientation angle. The right (left)-circularly-polarized light has points at the upper (lower) poles of the sphere. In this work, right circularly polarized light is defined as a clockwise rotation against the propagation direction of the light. Absorption of measured samples is indicated by the radius of the Poincaré sphere, i.e., it is a measure of light intensity. Here, we assume that the radius of the Poincaré sphere is always unity by normalizing the Stokes parameter, as the focus on polarization state rather than absorption. The Cartesian coordinates of the Poincaré sphere correspond to the Stokes parameters, S_1, S_2, and S_3 as shown in Fig. S2. The Stokes parameters are determined by the difference in the intensity of two different components of the light beam as follows: S_1 is between horizontal and vertical polarization, S_2 is between +45°

and −45° polarization, and S_3 is between right and left-circularly polarized light. For example, S_1 = 1 is horizontal linearly polarized light and S_1 = −1 is vertical linearly polarized light.

Next, we explain the evaluation method of θ and Δ using the Poincaré sphere representation. Before considering the general case, we assume the simple case where the initial state of light is $\mathbf{S} = (S_1, S_2, S_3)^t = (1, 0, 0)^t$ and the light passes through a quarter wave plate (QWP) with an angle of the slow optic axis of 45°, which has linear birefringence. In this situation, the Stokes parameter shows positive rotation with a phase shift of $\Delta = \pi/2$ about the slow optic axis [S5, S6]. Here, as the angle of the slow optic axis of the QWP is equal to $\mathbf{S} = (0, 1, 0)^t$, the polarization state of light passing through the QWP is varied to left-circular polarization ($\mathbf{S} = (0, 0, -1)^t$). This means that we can determine θ and Δ by changes in the Stokes parameter made by the measured object. For instance, as shown in Fig. S2, which shows the Stokes parameter with and without samples ($\mathbf{S}_\mathrm{I}$ and $\mathbf{S}_\mathrm{II}$, respectively), θ and Δ are determined using the

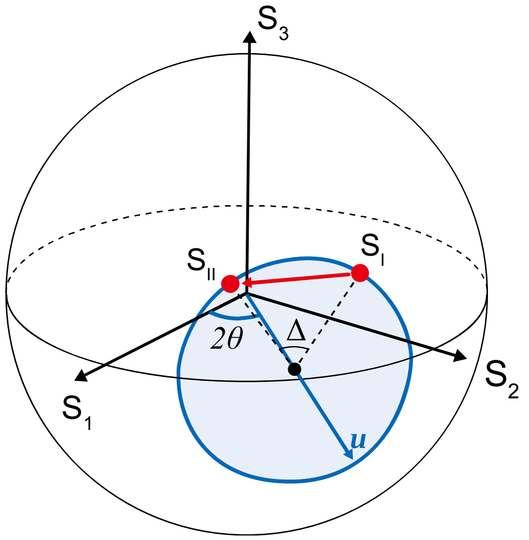

Figure. S2: Schematic of the angle of the slow optic axis θ and the phase difference Δ with changing polarization state on the Poincaré sphere.

Poincaré sphere representation as well as the simple case with a QWP. Moreover, it is found that when linearly polarized light with an identical orientation axis to the optic axis is incident on the sample, the polarization states do not change. To avoid this, we modulate the ellipticity of each frequency using the half wave plate.

Note that the above analysis is only valid in the case of linear birefringence being the dominant effect in the sample. If linear dichroism, circular birefringence, and circular dichroism of the sample are not negligible, the above analysis is not suitable and we should consider their effect on the Poincaré sphere representation. In this work, as the FKM samples show large linear birefringence and negligibly small linear dichroism between 0.2 and 0.3 THz as shown in Figs. 4a,b, the above analysis is valid for evaluating the birefringent properties of our samples.

Here, we briefly mention that our PS THz-TDS measurement system has the ability to determine both the optic axes and refractive indices along the optic axis of the samples, regardless of any angular difference between the orthogonal components of the terahertz wave and the optic axis of the sample. This means that we can analyze the optical responses of the samples along with their optic axes by rotating the two arbitrary orthogonal components of the terahertz waves such that they are parallel to their optic axes, though we always measure the x- and y-direction of terahertz electric fields.

Birefringent properties of various elastomers: To investigate the effect of CB on the birefringent properties, we measured various elastomer samples with and without CB. We prepared three elastomer samples (see Fig. S3a): a fluoroelastomer (FKM) (V-100, Togawa Rubber), a styrene-butadiene rubber (SBR) that includes CB with 31.4 wt% (Ukawa Rubber), and a natural rubber (NR) (AGS-14, Wakisangyo Co., Ltd). The NR

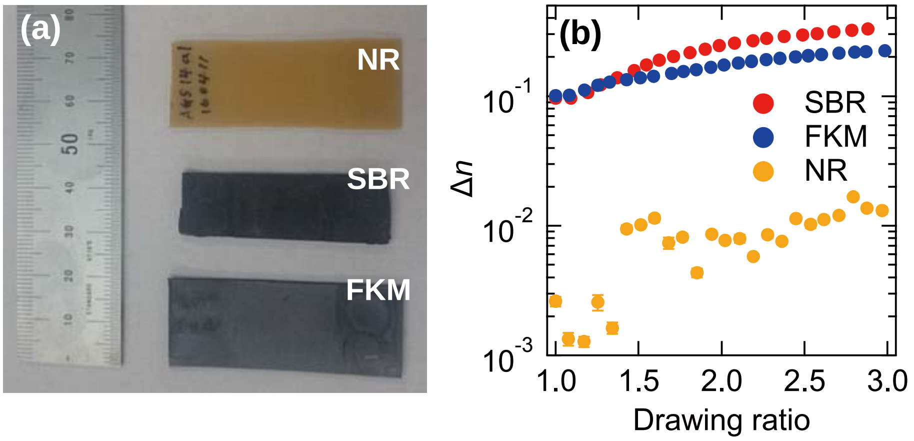

Figure. S3: (a) Photo and (b) drawing-ratio-dependent birefringence of FKM, SBR, and NR samples.

sample does not contain CB. Figure S3b shows the drawing ratio (DR) dependence of the birefringence for various elastomers. Samples containing CB (SBR and FKM) show a birefringence that is an order of magnitude larger compared to it without CB (NR). This indicates that the CB has a strong impact on the birefringent properties of the elastomer within the terahertz frequency region.

Procedures of Monte-Carlo (MC) simulation for calculating the macroscopic orientation: In order to investigate the relationship between external stress and the angle of the slow optic axis, we assume that the macroscopic orientation of anisotropic CB aggregates determines the angle of the slow optic axis. We calculate the macroscopic orientation of CB aggregates based on an MC simulation. We assume all anisotropic CB aggregates as uniform ellipsoids with unit length. One end of the ellipsoid is at the center of a unit sphere, with the orientation represented by the spherical angles Θ and ϕ on the sphere, as shown in Fig. S4. To show a random orientation of each ellipsoid with slight orientation, corresponding to our experimental

results, Θ and ϕ must be selected randomly as follows:

$$\begin{cases} \Theta = \cos^{-1}(z) \\ \phi = v \end{cases}$$

where z is a random number with normal distribution, a center value of 0, and a standard deviation σ, and v is a random number with uniform distribution in the range $[-\pi, \pi)$. We rotate Θ about the y-axis for setting the center angle of distribution to reproduce the experimental condition, for example, −9°, −52°, and −88°. Finally, we obtain the anisotropic orientational distribution of ellipsoids characterized by Θ and ϕ (see Fig. S5a).

Next, we consider the effect of uniaxial affine deformation of the sample on the anisotropic orientational distribution of ellipsoids. The uniaxial extension of the sample with a Poisson's ratio of 0.5 is described by the strain tensor, $\widehat{\Lambda}$;

$$\widehat{\Lambda} = \begin{pmatrix} \lambda^{-0.5} & 0 & 0 \\ 0 & \lambda^{-0.5} & 0 \\ 0 & 0 & \lambda \end{pmatrix},$$

where λ is DR. Here, we consider that the extended direction is parallel to the

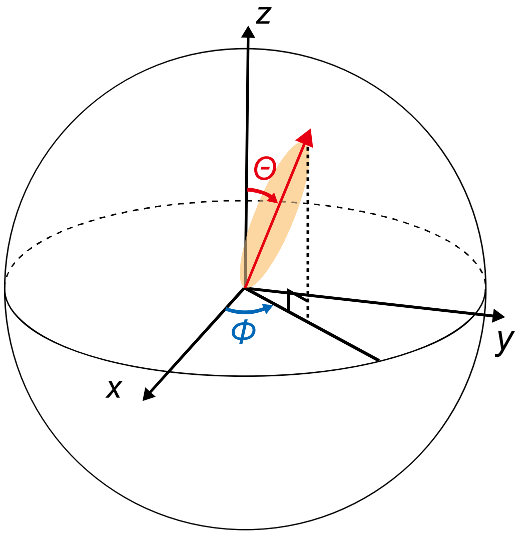

Figure. S4: Schematic of the orientation of the ellipsoids in the unit sphere.

z-direction. Under the uniaxial affine deformation λ, the orientation of the ellipsoids parallel to the stretched direction occurs as follows[S7]:

$$\Theta' = \tan^{-1}\left(\lambda^{-\frac{3}{2}}\tan\Theta\right),$$

where Θ' is the angle of the ellipsoids after the deformation. As the compression around the z-axis is isotropic, ϕ retains its initial value. The length of ellipsoids is unchanged by stretching, because we assume that the CB aggregates are rigid. Thus, we can calculate the orientational distribution of the ellipsoids at each λ by changing from Θ to Θ'. Figure S5a,b show the calculated orientational distribution of the ellipsoids before and after deformation using 3,000 samples and $\lambda = 3$. The center angle, Θ_0, and σ of the initial normal distribution are −9° and 0.21, respectively. This matches well with experimental results (see Fig. 6a).

Finally, we explain the calculation method of the macroscopic orientation based on the orientational distribution. The macroscopic orientation is typically characterized by a unit vector $\boldsymbol{n}$, the so-called director. Consider the example where one optic axis is parallel to $\boldsymbol{n}$. In the research field of the liquid crystals, $\boldsymbol{n}$ is usually determined by tensor order parameter $Q_{\alpha\beta}$ [S8], which is defined by,

$$Q_{\alpha\beta} = \left(\langle u_\alpha u_\beta \rangle - \frac{1}{3}\delta_{\alpha\beta}\right),$$

where u_α is the αth Cartesian coordinate of the unit vector for each ellipsoid, $\alpha, \beta = x, y, z$, $\delta_{\alpha\beta}$ is the Kronecker delta, and $\langle \cdots \rangle$ is the ensemble average. It is well established that after diagonalizing $Q_{\alpha\beta}$, the eingenvector corresponding to the largest positive eigenvalue represents $\boldsymbol{n}$. After deriving $Q_{\alpha\beta}$ with the orientational distribution calculated by the MC simulation and diagonalizing it, we can determine the angle of the slow optic axis through $\boldsymbol{n}$.

Figures S5a,b show the orientational distribution with $\Theta_0 = -9°$ and $\sigma = 0.21$ at different DRs on the unit sphere. The dots on the unit sphere represent the orientation of the ellipsoids. At DR = 1, the dots are widely spread over the sphere. At DR = 3, the dots aggregate around the z-axis. To evaluate the distribution of Θ, the histogram of the z coordinate for DR = 1 and 3 with 1,000,000 samples are plotted as shown in Figs. S5c,d. In contrast to the histogram for DR = 1, the z value concentrates in the vicinity of $z = \pm 1$ at DR = 3. The standard deviation of z at DR = 3 is estimated to be 0.02, corresponding to approximately 1.3°. This means that the ellipsoids are almost parallel

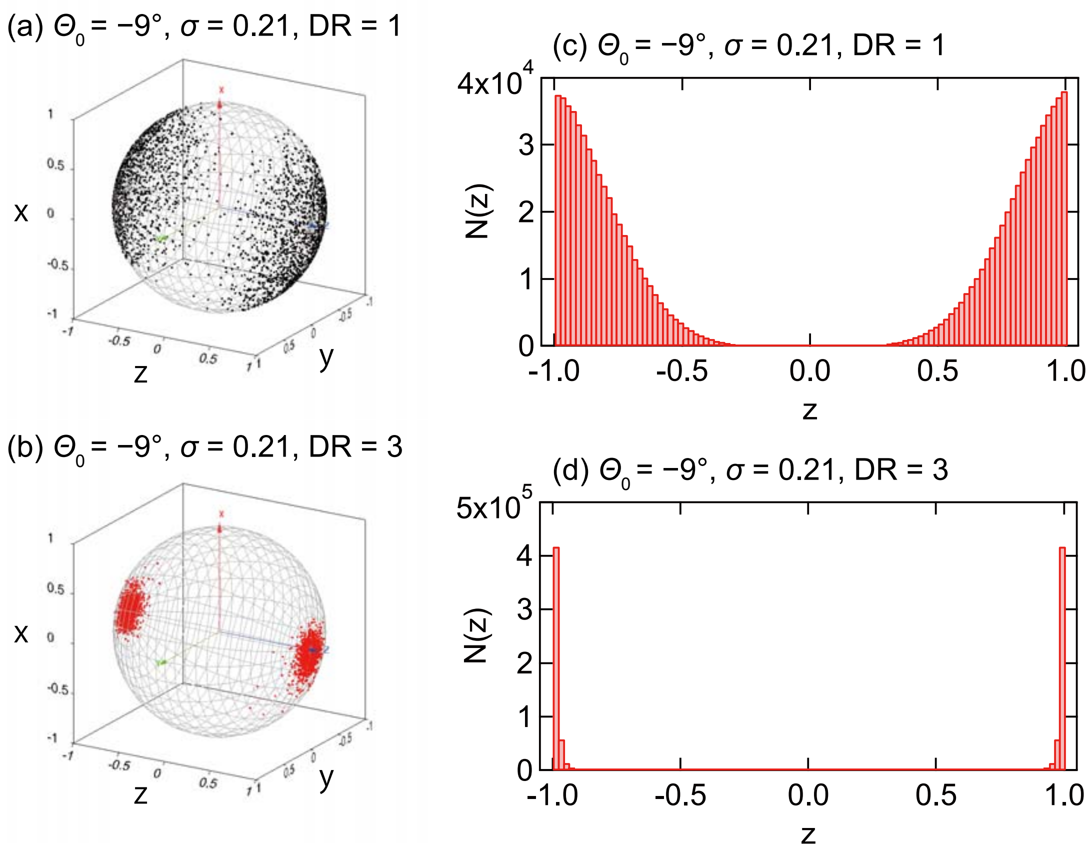

Figure. S5: Orientational distribution at DR = 1(a) and 3(b) on the unit sphere with $\Theta_0 = -9°$ and $\sigma = 0.21$. Each dot on the sphere corresponds to each orientational direction of the ellipsoid. (c) and (d) represent the histograms of the z coordinates, corresponding to the cases of (a) and (b), respectively.

to the stretched direction at DR = 3. Based on the simulation results, we fitted the dielectric function and the conductivity in Figs. 7a,b under the assumption of a fully oriented condition.

Figures S6a–c show the orientational distribution with $\Theta_0 = -88°$ and σ = 0.21 at DR = 1, 2.12, and 3, respectively. In the case where Θ_0 is far from 0°, the drawing ratio dependence of the angle of the slow optic axis shows characteristic behavior as shown in Fig. 6a. At DR = 2.12, the angle of the slow optic axis is evaluated to be −45°, whereas the birefringence is almost equal to zero in our experiment (see Fig. 6b). In this case, the distribution of orientations appears not to be isotropic, as shown in Fig. S6b. At DR = 3, although the angle of the slow optic axis is calculated to be almost 0, a part of the ellipsoid remains around Θ = 90°, which is in stark contrast to the result where Θ_0 is −9° (shown in Fig. S5b). These results are clearly shown in the histogram of the *z* coordinate with 1,000,000 samples as shown in Figs. S6d–f. Even at large DR conditions, a small number of the ellipsoids remain perpendicular orientation to the stretched direction.

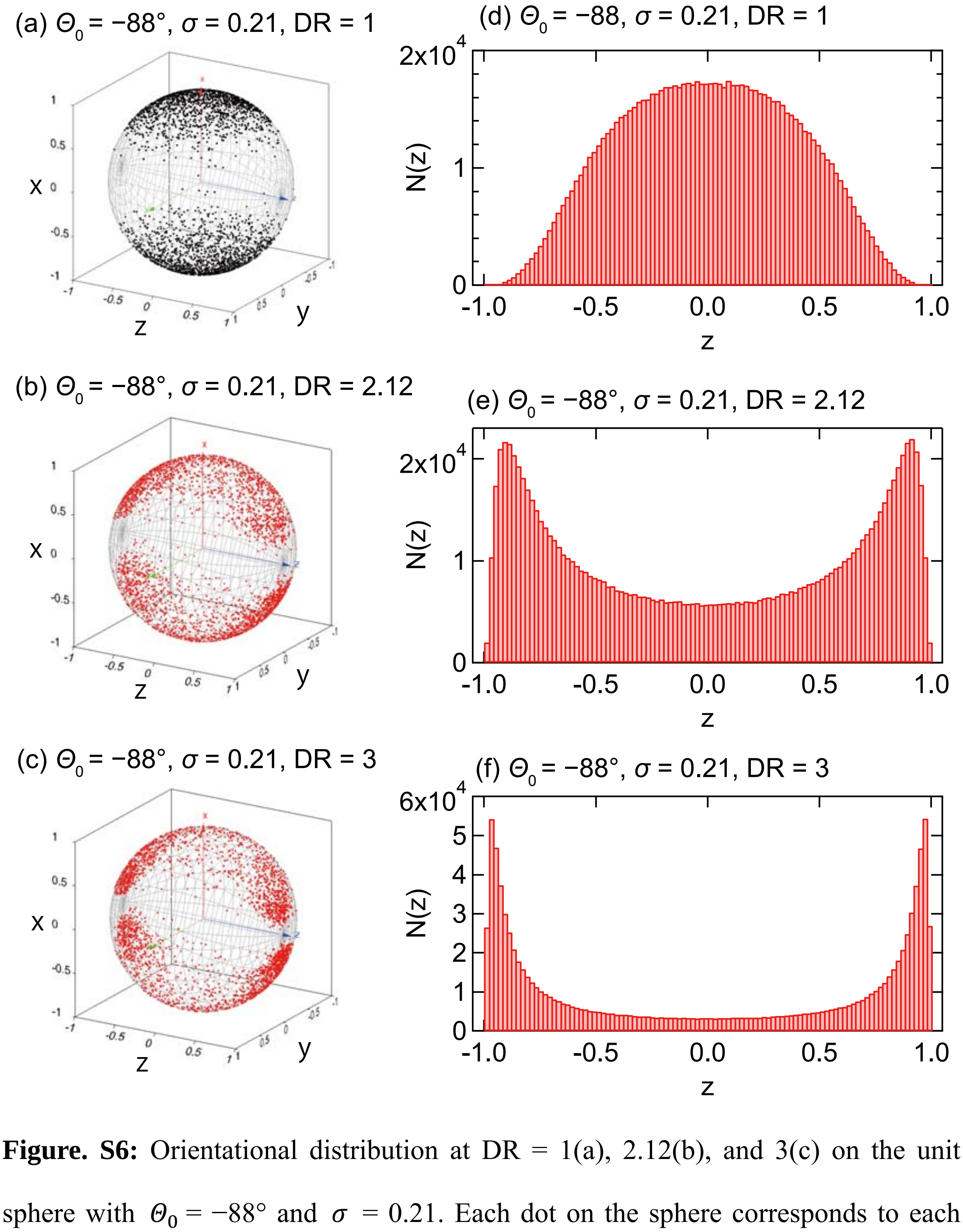

Figure. S6: Orientational distribution at DR = 1(a), 2.12(b), and 3(c) on the unit sphere with $\Theta_0 = -88°$ and $\sigma = 0.21$. Each dot on the sphere corresponds to each orientational direction of the ellipsoid. (d), (e), and (f) represent the histograms of the *z* coordinates, corresponding to the cases of (a), (b), and (c), respectively

References

S1. Morris, C. M., Aguilar, R. V., Stier, A. V. & Armitage, N. P. Polarization modulation time-domain terahertz polarimetry. *Opt. Express* **20**, 12303–12317 (2012).

S2. Castro-Camus, E. *et al*. Polarization-sensitive terahertz detection by multicontact photoconductive receivers. *Appl. Phys. Lett.* **86**, 254102 (2005).

S3. Born M.; Wolf E. *Principles of Optics: Electromagnetic Theory of Propagation, Interference and Diffraction of Light,* 7th ed. (Cambridge University Press, 1999).

S4. Bennett, J. M. *Handbook of Optics* Vol. 1*,* 2nd ed. (ed. Bass. M) ch. 5 (McGraw-Hill, 1995).

S5. Ramachandran, G. N. & Ramaseshan, S. Magneto-Optic Rotation in Birefringent Media – Application of the Poincaré Sphere. *J. Opt. Soc. Am.* **42**, 49–56 (1952).

S6. Jerrard, H. G. Transmission of Light through Birefringent and Optically Active Media: the Poincaré Sphere. *J. Opt. Soc. Am.* **44**, 634–646 (1954).

S7. Ahir, S. V. & Terentjev, E. M. Photomechanical actuation in polymer-nanotube composites. *Nat. Mater.* **4**, 491–495 (2005).

S8. de Gennes P. G. & Prost, J. *The Physics of Liquid Crystals*, 2nd ed. (Oxford University Press, 1993).
